# Recognizing and Preventing Overexposure to Methylmercury from Fish and Seafood Consumption: Information for Physicians

**DOI:** 10.1155/2011/983072

**Published:** 2011-07-13

**Authors:** Susan M. Silbernagel, David O. Carpenter, Steven G. Gilbert, Michael Gochfeld, Edward Groth, Jane M. Hightower, Frederick M. Schiavone

**Affiliations:** ^1^Stony Brook University, 167 Dana Hall, Stony Brook, NY 11794-5000, USA; ^2^University at Albany, State University of New York, 5 University Place, A217, Rensselaer, NY 12144, USA; ^3^Institute of Neurotoxicology and Neurological Disorders, 8232 14th Avenue NE, Seattle, WA 98115, USA; ^4^Robert Wood Johnson Medical School, University of Medicine and Dentistry of New Jersey, Piscataway, NJ 08854, USA; ^5^Groth Consulting Services, 75 Clifford Avenue, Pelham, NY 10803, USA; ^6^California Pacific Medical Center, 2100 Webster Street, San Francisco, CA 94115, USA; ^7^School of Medicine, Stony Brook University, 4-175 Health Sciences Center, Stony Brook, NY 11794-8430, USA

## Abstract

Fish is a valuable source of nutrition, and many people would benefit from eating fish regularly. But some people eat a lot of fish, every day or several meals per week, and thus can run a significant risk of overexposure to methylmercury. Current advice regarding methylmercury from fish consumption is targeted to protect the developing brain and nervous system but adverse health effects are increasingly associated with adult chronic low-level methylmercury exposure. Manifestations of methylmercury poisoning are variable and may be difficult to detect unless one considers this specific diagnosis and does an appropriate test (blood or hair analysis). We provide information to physicians to recognize and prevent overexposure to methylmercury from fish and seafood consumption. Physicians are urged to ask patients if they eat fish: how often, how much, and what kinds. People who eat fish frequently (once a week or more often) and pregnant women are advised to choose low mercury fish.

## 1. Introduction

All forms of mercury are toxic: elemental, inorganic, and organic forms. Methylmercury (MeHg) is the major organic form we are exposed to when we eat fish. All fish and shellfish contain some MeHg, but larger, longer-lived predatory fish generally have the highest levels. MeHg is particularly hazardous because it can cross the blood-brain barrier. Manifestations of MeHg poisoning are very variable and may be difficult to detect unless a test for blood or hair mercury is performed.

Exposure to elemental mercury in vapor form, for example, from broken thermometers or fluorescent light bulbs, can cause acute adverse effects. Inorganic mercury compounds can cause kidney toxicity, but exposure is uncommon except in certain occupational settings. This document focuses on MeHg exposure from fish and shellfish consumption. For information on other forms of mercury relevant to human health (e.g., from vaccines, silver-colored amalgams, and skin lightening creams) please see information from the Agency for Toxic Substances and Disease Registry [1].

In 2004, the Environmental Protection Agency (EPA) and the Food and Drug Administration (FDA) issued a joint advisory on fish consumption and MeHg [2]. The EPA/FDA advice is targeted to the higher risk populations of women of childbearing age, pregnant and nursing women, and young children (<6 years old). But fish consumers of all ages and genders who eat several meals of fish per week, or who regularly eat fish with higher levels of MeHg, are at risk of exceeding the EPA reference dose (RfD), a level of exposure that is prudent for all people to use as a guide to safe fish consumption. 

State health departments in most states have issued advisories about MeHg contamination of locally caught sport fish. Some state health departments include advice on commercially caught fish, but most do not. As a result, many consumers are unaware that MeHg in commercially caught fish can have health effects.  Table 1 identifies commercial fish with the highest MeHg levels.

Data from the National Health and Nutrition Examination Survey (NHANES) conducted by the Centers for Disease Control and Prevention (CDC) show that blood mercury levels are strongly correlated with fish consumption. Levels are higher among people with higher incomes, among ethnic groups that eat more fish, such as Native American, Asian and Pacific Island populations [3], and among residents of US coastal regions [4]. A recent New York City Department of Health & Mental Hygiene study estimated that almost 25% of adults in New York City and almost 50% of Asian New Yorkers have blood mercury levels above 5 *μ*g/L [5]. Nationally, about 7% of the NHANES sample exceeded that level [4]. The data illustrate there are identifiable, specific groups of people that are most vulnerable to overexposure to MeHg from fish consumption. With all of the public debate around the benefits and risks of fish consumption, we see informing physicians about MeHg in fish as an important way to reach the persons at greatest risk of overexposure to MeHg from fish and seafood consumption. 

## 2. How Does Mercury Get into Fish?

Mercury occurs in the environment both naturally and as a result of human activities. The largest source of mercury emissions is coal combustion for energy production (cement kilns and chlor-alkali plants are also sources). When coal is burned, elemental mercury and inorganic mercury compounds are released and can be carried long distances in the air before they fall out onto land or water bodies. In water and wetlands, inorganic mercury is transformed by microorganisms to a more toxic form, MeHg. MeHg biomagnifies in the aquatic food chain and larger predatory fish such as shark, swordfish, king mackerel, and certain species of tuna accumulate some of the highest levels.

## 3. What Happens in the Body When MeHg Is Consumed?

More than 95% of the MeHg consumed in fish is absorbed in the gastrointestinal tract and transported to the blood stream, whence it is distributed to all organs. It takes only about 30–40 hours for an ingested dose of MeHg to be completely distributed throughout the body. Some organs have a higher affinity for MeHg than others. It crosses the blood-brain barrier and accumulates in the brain, where it can damage the central nervous system. About 10% of the MeHg in the body is in the brain; there, it is slowly demethylated to inorganic mercury, which crosses the blood-brain barrier very poorly. 

Mercury—MeHg and demethylated—is gradually removed from the body, primarily via liver bile and feces, but some is also excreted in urine, sweat, and breast milk and some is stored in hair and nails. The MeHg level in blood is assumed to reflect the total amount in the body. The half-life of MeHg in blood is about 50–70 days in adults but can vary significantly; the half-life is longer in neonates and research suggests that genetic variation may account for additional differences. 

MeHg crosses the placenta, and levels in umbilical cord blood are about 1.7 times as high as the mother's blood levels. To keep fetal blood mercury below the EPA reference level of 5.8 *μ*g/L, the mother's blood level should thus not exceed 3.5 *μ*g/L [6]. The developing nervous system is known to be particularly vulnerable to MeHg; effects depend on both the dose and the timing of exposures. Prenatal exposure to MeHg can result in cognitive deficits [7], motor skill effects [8], attention deficits, language skill deficiencies [9, 10], and decreased learning capacity and memory [11, 12]. 

Fish consumption also has well-documented nutritional benefits that improve cognitive test performance in children of women who ate low-mercury fish during pregnancy. Mothers-to-be should therefore be encouraged to eat fish, but advised to choose only low-mercury varieties [9, 10].

Research has also shown positive cardiovascular effects of fish consumption, probably due to omega-3 fatty acids [13]. But recent studies have also linked negative cardiovascular effects with the MeHg exposure associated with fish consumption, including increased heart rates and blood pressure [14] and a greater risk of myocardial infarction [15–17]. MeHg may also affect immune system function [18]. The important message is that the benefits of eating seafood clearly exceed the risks from MeHg, as long as the fish consumed are mostly low in mercury [19–21].

## 4. How Can You Identify Patients with Health Effects from MeHg?

Clinical manifestations (see Table 2) vary with the degree and length of exposure, and symptoms may not appear for some length of time after high exposure begins. Symptoms can vary significantly from individual to individual. Some studies suggest that symptoms may emerge when the body's ability to compensate for the damage is depleted [22, 23]. Other research suggests that genetic variation, specific food interactions that effect mercury metabolism, and other characteristics of individuals influence the manifestation of symptoms [18, 24, 25].

Patients with chronic lower level exposure to MeHg can experience nonspecific health effects such as fatigue, difficulty concentrating, hair thinning, muscle and joint pain, sleep disturbance, and gastrointestinal upset. Classical signs and symptoms of higher level MeHg exposure include (in order of typical appearance of symptoms) numbness and tingling around the mouth, numbness and tingling in hands and feet, clumsy gait or difficulty walking (ataxia), slurred speech, visual field constriction, coma, convulsions, and death. Patients with very high MeHg exposure may also exhibit symptoms described for lower exposures. 

Multiple research studies and personal observations by the authors of this document indicate that individual patients vary widely in sensitivity to MeHg toxicity. The milder symptoms have been seen in some patients at relatively low blood mercury levels [15, 26–28]. A review of 24 cases of symptomatic MeHg poisoning found a range from 7 to 125 *μ*g/L blood mercury, and the majority of cases had levels below 40 *μ*g/L [27].  Table 3 briefly describes some cases of MeHg exposure. People vary in their susceptibility to mercury and not everyone will experience negative health effects. Patients at greatest risk of developing MeHg poisoning are those who eat fish often and who prefer higher-mercury seafood varieties such as swordfish or tuna [29]. Thus it is important that health care professionals ask patients about their diet in order to make the connection between MeHg exposure and seafood consumption.

## 5. Laboratory Tests and Their Interpretation

A blood analysis should be done for patients with suspected elevated MeHg exposure from fish and shellfish consumption. A hair sample may also be analyzed; the level in hair reflects longer-term exposure and helps distinguish organic (methyl- or ethyl-) mercury exposure from inorganic or elemental mercury exposure. Urine tests primarily reflect inorganic and elemental mercury exposures. In general a low urine mercury test (<10 *μ*g/L) in combination with elevated blood (>5 *μ*g/L) or hair (>1 *μ*g/g) mercury points to MeHg exposure from seafood consumption. While most clinical analyses of blood, nail clippings, or hair are for total mercury, almost all mercury present is in the form of MeHg.

**Blood Test**

While the EPA has defined criteria for excessive blood mercury in women of childbearing age, there are no generally recognized guidelines for acceptable blood mercury in the rest of the population. Geometric mean blood levels in the USA based on NHANES data are <1 *μ*g/L for those age 29 and under and about 1 *μ*g/L for those 30 and older. Blood mercury levels tend to increase with age and peak in the 5th or 6th decade, depending on race and ethnicity [30]. The Centers for Disease Control and Prevention define the laboratory criteria for diagnosis of excessive mercury exposure as a blood level above 10 *μ*g/L [31]. Some state departments of health, including New York [32], require laboratories that measure blood mercury to report levels above 5 *μ*g/L to the state heavy metals registry. The authors of this document believe that a blood mercury level above 5 *μ*g/L calls for counseling of patients with regard to fish consumption emphasizing low mercury species.

Blood mercury tests reflect recent exposures as well as chronic accumulation. Blood labs instruct patients not to eat seafood for three days before a mercury test, but patients should be advised to follow their normal diet prior to testing.

**Hair Test**

Most people have hair mercury levels well below 1 *μ*g/g (ppm), the EPA reference level. Neuropsychological functional deficits have been reported in adults with an average hair level of 4.2 ppm [33], while prenatal neurodevelopmental effects have been associated with maternal hair levels of 1.2 ppm or higher [9]. A 2009 literature review looking at neurodevelopmental effects on the fetus estimated that a lowest observable adverse effect level in maternal hair might be as low as 0.3 *μ*g/g [34], thus supporting a precautionary approach that includes counseling those planning to be pregnant, or who already are pregnant, to choose low mercury fish.

## 6. Recommended Action for Those with High Blood or Hair Mercury

The primary advice for patients with MeHg poisoning is to stop eating fish temporarily or shift to very low-mercury fish. Once their blood mercury has declined to a lower level (<5 *μ*g/L) and symptoms have resolved, low-mercury fish and shellfish can be reintroduced to the diet. 

Chelation can be a valuable medical intervention for *inorganic *mercury poisoning, but it poses its own risks and, except in rare cases, is not generally warranted for patients with elevated MeHg from fish consumption [11, 35]. Some practitioners mistakenly use a DMSA or DMPS provocation challenge when they test a patient's urine for mercury. This gives highly misleading results that overestimate, sometimes seriously, a patient's mercury exposure. There is also individual variability in response to chelation challenge or treatment.

## 7. Prevention and Risk Communication

To make healthy fish consumption choices, consumers need to know which fish are lowest in contaminants and higher in omega-3 fatty acids (see Table 4 for fish guidance considering both MeHg and PCB contaminant levels). Fish is a good source of protein and is low in saturated fat. Advise fish eaters to choose low-contaminant, high omega-3 fatty acid varieties, and to limit consumption of higher mercury fish. Pregnant women, women who are breastfeeding, women who plan to become pregnant within a year, and children less than 12 years old should eat *only *low-mercury fish. 

Mercury accumulates in fish muscle and levels are not reduced by cooking. Persistent organic pollutants like PCBs accumulate in fat, and exposures can be decreased by removing skin and fatty tissue and letting fat drip off during cooking [36].

## 8. How Much Fish Can You Eat?

A level of mercury consumption with no adverse effects has not been established. The EPA RfD is a guideline for acceptable daily exposure based on the best evidence available in 1999, but more recent studies, as noted earlier, suggest that more caution is justified, especially for pregnant and nursing women and children. The RfD assumes that prenatal cognitive effects are the most critical hazard and aims to prevent those effects. Given its narrow basis, the RfD is at best a guideline for acceptable exposure in other populations. Recent studies suggest adult cardiovascular health is sensitive to low levels of MeHg [37]. Neurobehavioral functions in adults can be impaired at doses similar to those that cause prenatal effects [28, 33]. Two recent epidemiological studies have found both beneficial effects of fish nutrition and adverse effects of MeHg exposure on prenatal cognitive development at MeHg doses near or below the RfD, that is, doses an order of magnitude lower than those recognized as harmful in 1999 [10, 12, 38]. To be on the safe side, physicians are advised to encourage frequent fish eaters to try to keep their MeHg exposure *below* the RfD. This approach is especially important for pregnant women, but given the uncertainties, it may prudently be applied to other patients as well. 

How many fish or shellfish meals are advised per week depends on *how often* one eats fish, a person's *body weight*, the *portion size*, the *mercury content* of the fish choice, and *individual health considerations* such as pregnancy status. Individual patients can also vary widely in their inherent susceptibility to toxic effects. Portion size is an important factor in exposure for many Americans in particular because we tend to eat large portions. According to the US Department of Agriculture, a serving is 2-3 ounces per meal for a recommended total daily total protein amount of 6-7 ounces of lean meat or fish, depending on your energy requirements [39]. Serving sizes at home and in restaurants are often larger [40] and thus should be factored into exposure considerations. Portion sizes for children should be approximately one ounce for every twenty pounds of body weight. For example, a serving for a 45 pound child would be about 2 ounces.

Adults who eat a very high MeHg fish such as swordfish or shark even as infrequently as once a month will generally exceed the EPA reference dose.

Many people consume both commercial and sport fish and thus total fish consumption should be considered. Check state public health departments for local fish advisory information (links are included in the resources section).

## 9. Other Contaminants of Concern

Fish also contain persistent organic pollutants (POPs) such as PCBs. Research suggests that POPs have their own negative health effects that may offset some of the benefits of fish consumption [41–44]. Fat from pork, beef, and chicken also contains POPs, usually at lower levels than in fish. But most Americans eat much more beef, pork, and chicken than fish, so fish is not the largest dietary source of POPs for most consumers.

Many people take fish oil supplements to obtain the benefits from the omega-3 fatty acids present in fish. Some brands of fish oil supplements specify that they have been molecularly distilled or purified to remove contaminants and contain no detectable mercury. As MeHg binds to proteins, it is not present in the fat (oil). However, PCBs and other persistent organic pollutants and halogenated natural products do accumulate in fat and may contaminate supplements. The issue is complicated by the presence of multiple brands of fish oils with varying sources of fish that contain varying contamination levels, as well as varying levels of purification. Lack of any government standards as to acceptable levels of contamination further complicates the issue. Until more is understood, the most prudent approach is to consume a variety of low mercury fish—generally smaller, nonpredatory fish—in order to obtain the health benefits. If supplements are desired, those derived from small, cold water fatty fish such as anchovies, sardines, and mackerel are reported to have lower levels of organic contaminants [45].

For those who wish to consider both MeHg and POPs in their fish choices, some advice does include both types of contaminants for commonly eaten commercial and sport fish (see Washington State Department of Health and Connecticut State Departments of Public Health as well as the Environmental Defense Fund and Sea Web Kid Safe Seafood web sites; web site addresses are in the Resources section).

## 10. Resources

### 10.1. Online Tools for Guiding Low MeHg Fish Choices

Several organizations offer easy to use online calculators that estimate safe fish intakes based on the consumer's body weight and the fish selected. These calculators are designed to keep exposure below the EPA RfD. MeHg levels in seafood vary by geographical location with certain species such as tuna showing more variability [46], therefore we suggest that you use online tools more for general guidance to help understand fish MeHg levels, and not as an absolute index of MeHg content. The most prudent advice is to eat low-mercury fish. 

GotMercury.org (http://www.gotmercury.org/) and the Natural Resources Defense Council (http://www.nrdc.org/health/) both offer easy to use calculators that estimate safe intakes based on the EPA RfD and can help consumers make healthy seafood choices. Beware of some other online calculators that understate mercury risks. (e.g., the Howmuchfish.com website offers an on-line calculator that tells users they can consume ten times more methylmercury than the EPA RfD would indicate.)

Another option is an iPhone application called Fish4Health developed by researchers at Purdue University. Fish4Health lists seafood choices organized by mercury contamination levels and allows you to select the fish and the quantity you eat. It calculates and reports daily mercury intake and associated omega-3 fatty acid intake and keeps a log for you, letting you know if you are eating sufficient omega-3 fatty acids and if you exceed the EPA RfD for mercury. The application can be used by all seafood eaters who want to keep their mercury exposures below the EPA reference dose even though its target audience is pregnant and nursing women. (http://fn.cfs.purdue.edu/fish4health/iPhoneApp.html).

### 10.2. Online Seafood Advice

The *Environmental Defense Fund* Seafood Selector includes recommendations on how many meals one can eat of specific fish each month and stay below the EPA RfD for mercury. It also considers persistent organic pollutants and thus offers more broadly protective guidance than the joint FDA/EPA mercury advice. The EDF site also includes a sushi guide: http://www.edf.org/page.cfm?tagID=1540. 

*Sea Web Kid Safe Seafood* considers MeHg and persistent organic pollutants and the advice is geared specifically for children: http://www.kidsafeseafood.org/.

The *Mercury Policy Project (MPP)* sorts top selling commercial seafood varieties into six categories by mercury content, offering finer distinctions on mercury content for those who seek the lowest-mercury fish: http://mercuryfactsandfish.org/wpcontent/uploads/2010/02/FishAndShellfishByMercuryLevel.pdf.

### 10.3. Government Information Sources

The oversight of commercial and sport fish and resulting consumption advice is handled by separate government agencies. State, tribal and local governments are responsible for local sport fish advisories and the Food and Drug Administration governs the sale of commercial fish. Although most fish consumed in the USA is commercial fish, only some State Departments of Health offer advice that consider both sport and commercially caught fish (e.g., see Washington (http://www.doh.wa.gov/ehp/oehas/fish/default.htm) and Connecticut (http://www.ct.gov/dph/cwp/view.asp?a=3140&q=387460). Fish advisories for various states can be accessed through this EPA site: http://www.epa.gov/waterscience/fish/states.htm. 

The *Environmental Protection Agency* (EPA) monitors mercury in the environment and regulates industrial releases. The EPA site also contains information on fish consumption. EPA issued a joint advisory with the FDA in 2004: http://www.epa.gov/waterscience/fish/advice/index.html. 

Background information on the EPA FDA joint advisory on mercury in fish: http://www.epa.gov/fishadvisories/advice/factsheet.html.

EPA funded educational video modules for health professionals on the risks and benefits of fish consumption: http://www.fish-facts.org. 

The *Food and Drug Administration* (FDA) is the agency responsible for the safety of commercial seafood. The FDA website offers information about MeHg, including the FDA database on mercury levels in different categories of fish and shellfish: http://www.fda.gov/Food/FoodSafety/Product-SpecificInformation/Seafood/FoodbornePathogensContaminants/Methylmercury/ucm115662.htm. 

The *Agency for Toxic Substances and Disease Registry* provides data and educational resources on all forms of mercury in the environment (http://www.atsdr.cdc.gov/tfacts46.html).

## Figures and Tables

**Table 1:** Fish with highest MeHg Contamination.

King mackerel
Tilefish (Gulf of Mexico)
Tuna (Bluefin, Bigeye)
Shark
Swordfish

Data derived from FDA, WA DOH, CT DPH, and EDF websites (links in resources section).

**Table 2:** Signs and symptoms of MeHg poisoning.

Lower level exposures
Sleep disturbance
Headache
Fatigue
Difficulty concentrating
Depression
Memory loss
Diminished fine motor coordination
Muscle and joint pain
Gastrointestinal upset
Hair thinning
Heart rate disturbance
Hypertension
Tremor
Numbness or tingling around the mouth

Highest level exposures
Numbness or tingling in hands and feet
Clumsy gait, difficulty walking (ataxia)
Slurred speech
Tunnel vision
Diminished visual acuity

**Table 3:** Cases of MeHg poisoning.

A 40-year-old lawyer who ate fish three or four times a week, primarily sea bass, could not sleep and lost his ability to concentrate. His hair contained 13 ppm mercury and his blood level was 58 *μ*g/L.

A middle-aged sales manager ate fish eight or nine times a week, usually choosing tuna, swordfish, halibut, or sea bass. She experienced chronic fatigue, muscle aches, memory and concentration loss, and thinning of hair. When diagnosed, her blood mercury level was 76 *μ*g/L.

A 66-year-old guitarist experienced a loss of fine motor coordination that affected her ability to play her instrument. She also had muscle weakness, thinning hair, and hand tremors. She had been eating swordfish and tuna steaks four to five times a week. Her blood mercury was 38 *μ*g/L.

A 64-year-old anthropologist who ate fish nine times a week, often choosing tuna, swordfish, sea bass, and halibut, suffered from chronic fatigue, headaches, memory loss and, hair loss. Her blood mercury level at diagnosis was 21 *μ*g/L.

A 10-year-old boy who had always been an “A” student began having problems concentrating and completing assignments in school. He lost his ability to catch a ball and developed hand tremors. He had eaten a can of tuna every day for a year. His blood mercury level was above 60 *μ*g/L.

Cases excerpted from Groth [27].

**Table 4:** Choose wisely.

Lowest contaminant levels
Anchovies^*☺*^
Arctic char
Atlantic mackerel (not king mackerel)^*☺*^
Catfish (U.S. farmed)
Cod
Haddock
Herring^*☺*^
Perch
Pollock (fish sticks)
Salmon (wild)^*☺*^
Sardines^*☺*^
Shellfish (oysters (Pacific^*☺*^), shrimp, clams, mussels, scallops)
Tilapia
Tuna (Skipjack/“chunk light”, not yellowfin)
Trout (Rainbow, farmed)^*☺*^

Medium to high contaminant levels
Black sea bass^*☺*^
Grouper
Halibut
Lobster
Mahi mahi
Orange roughy
Rockfish/red snapper
Sablefish/black cod^*☺*^
Salmon (farmed)^*☺*, ∞^
Spanish mackerel^*☺*^
Tuna (albacore^*☺*^/“white”, yellowfin/ahi)

Highest contaminant levels
Bluefish^∞^
Croaker (White/Pacific)^ ∞^
Eel^∞^ (American, European; not Conger eel)
King Mackerel
Tuna (Bluefin^∞^, Bigeye)
Shark
Swordfish
Tilefish (Gulf of Mexico, not Atlantic)
Weakfish/Seatrout^∞^

^*☺*^A good source of omega-3 fatty acids.

^∞^May contain harmful PCB levels.

Data derived from FDA, WA DOH, CT DPH, and EDF websites (links in resources section).
